# ERBB3 Positively Correlates with Intestinal Stem Cell Markers but Marks a Distinct Non Proliferative Cell Population in Colorectal Cancer

**DOI:** 10.1371/journal.pone.0138336

**Published:** 2015-09-14

**Authors:** Thierry Jardé, Lisa Kass, Margaret Staples, Helen Lescesen, Peter Carne, Karen Oliva, Paul J. McMurrick, Helen E. Abud

**Affiliations:** 1 Department of Anatomy and Developmental Biology, Monash University, Wellington Rd., Clayton, Victoria, Australia; 2 Centre for Cancer Research, Hudson Institute of Medical Research, Clayton, Victoria, Australia; 3 Cabrini Institute, Cabrini Health, Malvern, Victoria, Australia; 4 Department of Surgery, Cabrini Monash University, Malvern, Victoria, Australia; University Claude Bernard Lyon 1, FRANCE

## Abstract

Several studies have suggested ERBB3/HER3 may be a useful prognostic marker for colorectal cancer. Tumours with an intestinal stem cell signature have also been shown to be more aggressive. Here, we investigate whether ERBB3 is associated with intestinal stem cell markers in colorectal cancer and if cancer stem cells within tumours are marked by expression of ERBB3. Expression of ERBB3 and intestinal stem cell markers (LGR5, EPHB2, CD44s and CD44v6) was assessed by qRT-PCR in primary colorectal tumours (stages 0 to IV) and matched normal tissues from 53 patients. The localisation of ERBB3, EPHB2 and KI-67 within tumours was investigated using co-immunofluorescence. Expression of ERBB3 and intestinal stem cell markers were significantly elevated in adenomas and colorectal tumours compared to normal tissue. Positive correlations were found between ERBB3 and intestinal stem cell markers. However, co-immunofluorescence analysis showed that ERBB3 and EPHB2 marked specific cell populations that were mutually exclusive within tumours with distinct proliferative potentials, the majority of ERBB3+ve cells being non-proliferative. This pattern resembles cellular organisation within normal colonic epithelium where EPHB2 labelled proliferative cells reside at the crypt base and ERBB3+ve cells mark differentiated cells at the top of crypts. Our results show that ERBB3 and intestinal stem cell markers correlate in colorectal cancers. ERBB3 localises to differentiated cell populations within tumours that are non-proliferative and distinct from cancer stem cells. These data support the concept that tumours contain discrete stem, proliferative and differentiation compartments similar to that present in normal crypts.

## Introduction

Colorectal cancer is one of the most commonly diagnosed and lethal cancers worldwide, with more than 1.2 million new cases and 0.6 million deaths estimated in 2008 [1]. Therapeutic strategies that include surgical resection coupled to chemotherapy are relatively efficient in treating the whole tumour mass. However, many cancers will re-occur within months or years. Disease relapse has been suggested to be due to chemotherapy resistant cancer stem cells that disseminate prior to tumour resection. These multipotent cells are then able to give rise to a new tumour, leading to cancer recurrence and metastasis. Recent advances in colorectal cancer have led to the characterisation of intestinal stem cell markers, including LGR5, EPHB2, and CD44 [2–4]. In normal colonic epithelium, these markers are restricted to the base of crypts where stem cells reside. Higher expression levels of these markers are found in colorectal cancer compared to adjacent normal tissue and LGR5 expression positively correlates with the number of lymph node metastases [4–7]. Colorectal cancer patients with high expression of an intestinal stem cell gene signature, including LGR5 and EPHB2, have a 10-times higher risk of relapse compared to patients with low levels [8]. Understanding the signalling pathways and molecules regulating cancer stem cells is required to develop robust prognostic approaches and new therapeutic strategies.

The ERBB family of receptor tyrosine kinases, also known as HER receptors, consists of four members–ERBB1/HER1, ERBB2/HER2, ERBB3/HER3 and ERBB4/HER4. These receptors are key modulators of normal growth and development and their dysregulation has been implicated in the initiation and progression of cancers [9, 10]. Antibody-based therapies directed against ERBB1 or ERBB2, aimed at decreasing their signal transduction capabilities, have demonstrated clinical benefits in the treatment of several tumours [11]. Due to the absence of an active intracellular tyrosine kinase domain, ERBB3 was disregarded for several years as a cancer target [12]. However, ERBB3 has been recently proposed to be involved in acquired resistance to therapies and has emerged as a potential therapeutic target leading to the development of multiple anti-ERBB3 antibodies [13]. Although the precise function of ERBB3 in colorectal cancer is not fully understood, several studies suggest that ERBB3 is disrupted in tumours. For instance, ERBB3 somatic mutations have been identified in 11% of colon cancers and several studies have shown that ERBB3 is expressed in 36–89% of colorectal cancers [14–20]. Some studies have suggested that increased ERBB3 protein expression in colon cancer is also associated with decreased patient survival [20, 21]. *In vitro*, ERBB3 knockdown decreased cell proliferation, induced apoptosis and blocked migration of colon cancer cells [21]. A similar mechanism was observed *in vivo* where ERBB3 knockdown delayed tumour growth [14]. Although these data are clearly indicative of an oncogenic potential for ERBB3, it is currently unknown whether ERBB3 regulates the colorectal cancer stem cell pool that is suggested to drive tumour initiation and tumour recurrence [8, 22].

## Materials and Methods

### Patient recruitment and tissue collection

Fifty three patients with primary adenomas (n = 4) or adenocarcinomas (n = 49) underwent surgery in 2012/ 2013 at Cabrini Hospital (Malvern, Victoria). The study was approved by the Cabrini Human Research Ethics Committee. All patients provided written informed consent. Tumour specimens and matched normal colon tissues were freshly stored in RNAlater (Qiagen) for RNA extraction and fixed in 4% paraformaldehyde for tissue expression analysis.

### Cell culture

LIM1215, LIM1899, LIM2405, LIM2550, CACO2 and SW480 human colorectal cancer cell lines were used in this study (were supplied as a gift from The Ludwig Institute for Cancer Research, Parkville, Australia)[23–26]. Cells, except CACO2 cells, were routinely passaged in RPMI 1640 containing 10% foetal calf serum (Gibco), penicillin/streptomycin (Gibco) and glutamax (Gibco). CACO2 cells were maintained in DMEM containing 20% foetal calf serum (Gibco), penicillin/streptomycin (Gibco) and glutamax (Gibco). Cells were cultured in a humidified atmosphere of 5% CO2 at 37°C.

### RNA extraction, cDNA preparation and quantitative RT-PCR

Tissues obtained from resected colorectal cancers and matched normal tissue were homogenised and total RNA extracted using TRIzol reagent (Life Technologies) and RNeasy mini kit (Qiagen). Total RNA was extracted from colorectal cancer cells from the 6 cell lines using the RNeasy mini kit (Qiagen). RNA was reverse transcribed using the QuantiTect Reverse Transcription kit (Qiagen). Quantitative reverse transcriptase polymerase chain reaction (qRT-PCR) was performed using Brilliant II SYBR Green QPCR Master Mix (Agilent technologies). Triplicate samples were analysed on a LightCycler 480 machine (Roche Diagnostics). Gene expression levels were calculated using the 2^-DDCt^ method using the geometric mean of 2 housekeeping genes. β-ACTIN and β-2-MICROGLOBULIN as normalisers because they generated the best score when comparing carcinomas with normal mucosa [27, 28]. Primers are listed in S1 Table.

### Immunofluorescence

Fixed tissues were paraffin embedded and cut into 4 μm sections. Slides were deparaffinised in xylene, rehydrated in graded alcohols and incubated in citrate buffer solution (pH = 6) for 10 minutes in a pressure cooker.

Colorectal cancer cells from the 6 different cell lines were cultured for 4 days on Poly-L-lysine coated slides, washed with PBS and fixed with ice-cold acetone for 10 minutes.

Both tissue sections and colorectal cancer cell line slides were blocked with CAS block (Life Technologies) for 1 hour at room temperature before incubation overnight at 4 degrees with primary antibodies, EPHB2 (R&D, AF467, 1/50), ERBB3 (Abcam, ab93739, 1/200), KI-67 (DAKO, M7248, 1/50), MUC2 (Abcam, ab11197, 1/200) and P-H3 (Cell signaling, 9706S, 1/100). Slides were washed and then exposed to Alexa Fluor 488 donkey anti-goat IgG, Alexa Fluor 568 donkey anti-mouse IgG or Alexa Fluor 637 donkey anti-rabbit IgG (Invitrogen, 1/500) for 1 hour and counterstained with DAPI. Fluorescent images were taken on a Nikon C1 confocal microscope (Nikon, Japan). Illumination intensity, exposure, offset and gain settings were maintained between samples. The distribution of EPHB2 and ERBB3 in tumour cells was quantified by taking six representative images per tumour (n = 15 tumours). Around 3500 cells per tumour were manually counted using FIJI image analysis cell counter software. The quantification of P-H3+ cells and their localisation in EPHB2 or ERBB3 cell populations was conducted using the Aperio FL multiplexing immunofluorescence slide scanner. Fluorescent images of entire tissue sections (n = 8 tumours) were taken and all the P-H3 cells were quantified (around 100–200 P-H3+ cells per tumour) and localised within the different cell populations.

### Statistical analyses

Fold changes between tumour and matched normal samples were calculated as the ratio of gene expression in tumour versus normal. Because absolute gene expression levels were not normally distributed, non-parametric tests were used. Associations between gene expression were assessed using Wilcoxon matched-pairs sign-rank tests. To test the significance of fold changes, we performed one sample, two-sided t-tests of the natural logarithm of the fold changes = 0. Associations were assessed using Spearman rank correlations. Linear regression modelling, using the logarithm of the marker fold changes as the dependent variable, were used to investigate associations between marker expression and tumour characteristics. Tumour stage, T, N and M stages and tumour grade were included as variables with coefficients considered significant if the p-value was less than 0.05.

## Results

### Expression of ERBB3 and intestinal stem cell markers are elevated in colorectal cancers compared to matched normal tissues

The study population consisted of 53 patients (Patient characteristics in S2 Table). Intestinal lesions were characterised as pre-cancerous in 4 patients and cancerous in 49 patients. Expression of all markers, as assessed by qRT-PCR, was detected in all normal and tumour tissues.

**Fig 1:**
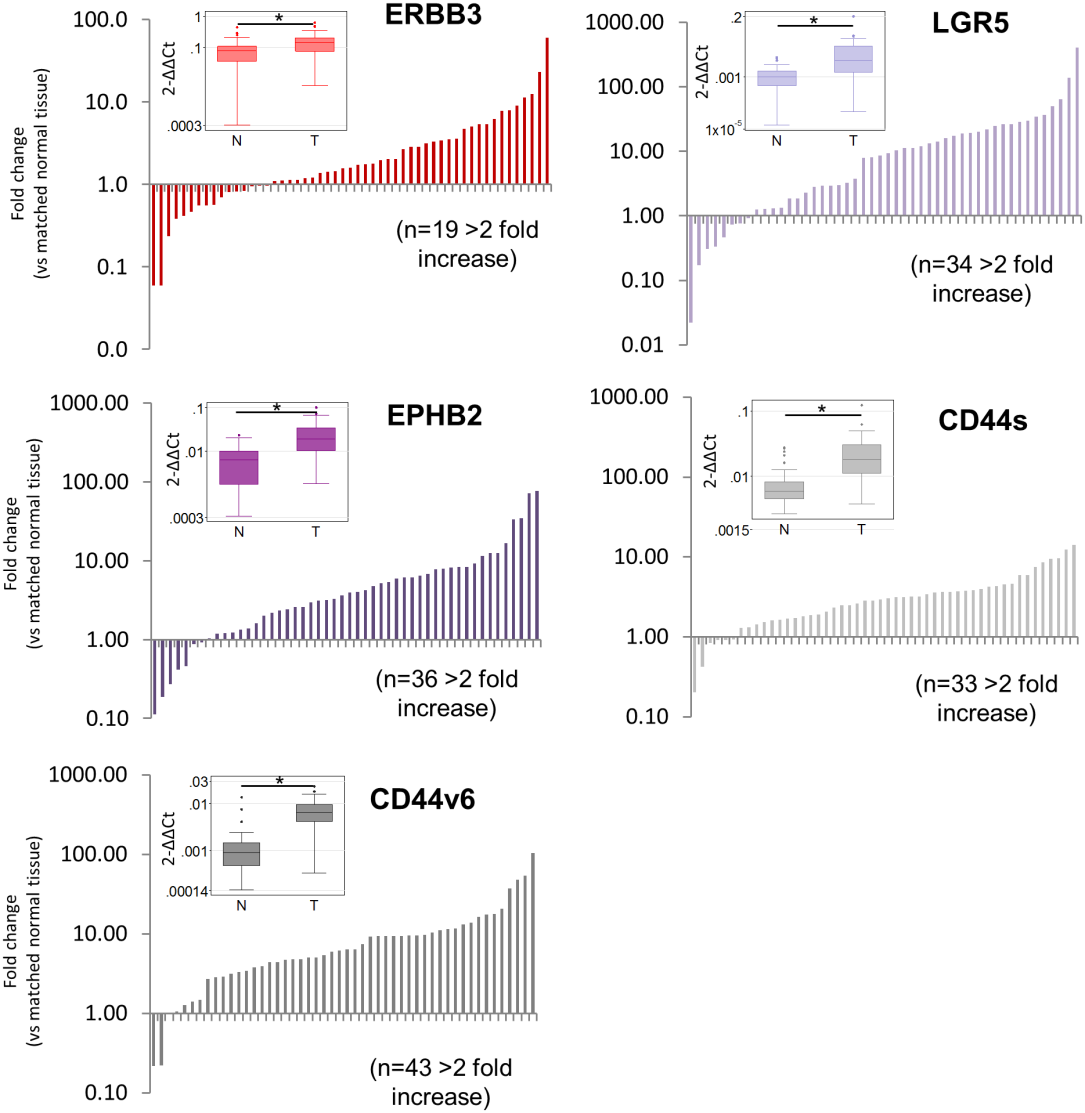
ERBB3 and intestinal stem cell markers LGR5, EPHB2, CD44s and CD44v6 are over-expressed in colorectal cancers. Quantitative real-time PCR results are expressed relative to matched normal tissue for each adenocarcinoma (n = 50). Inserts show boxplots comparing the absolute values of gene expression in normal (N) and tumour (T) tissues. * Significant difference compared to control tissues (Wilcoxon sign rank test, p<0.001).

**Fig 2:**
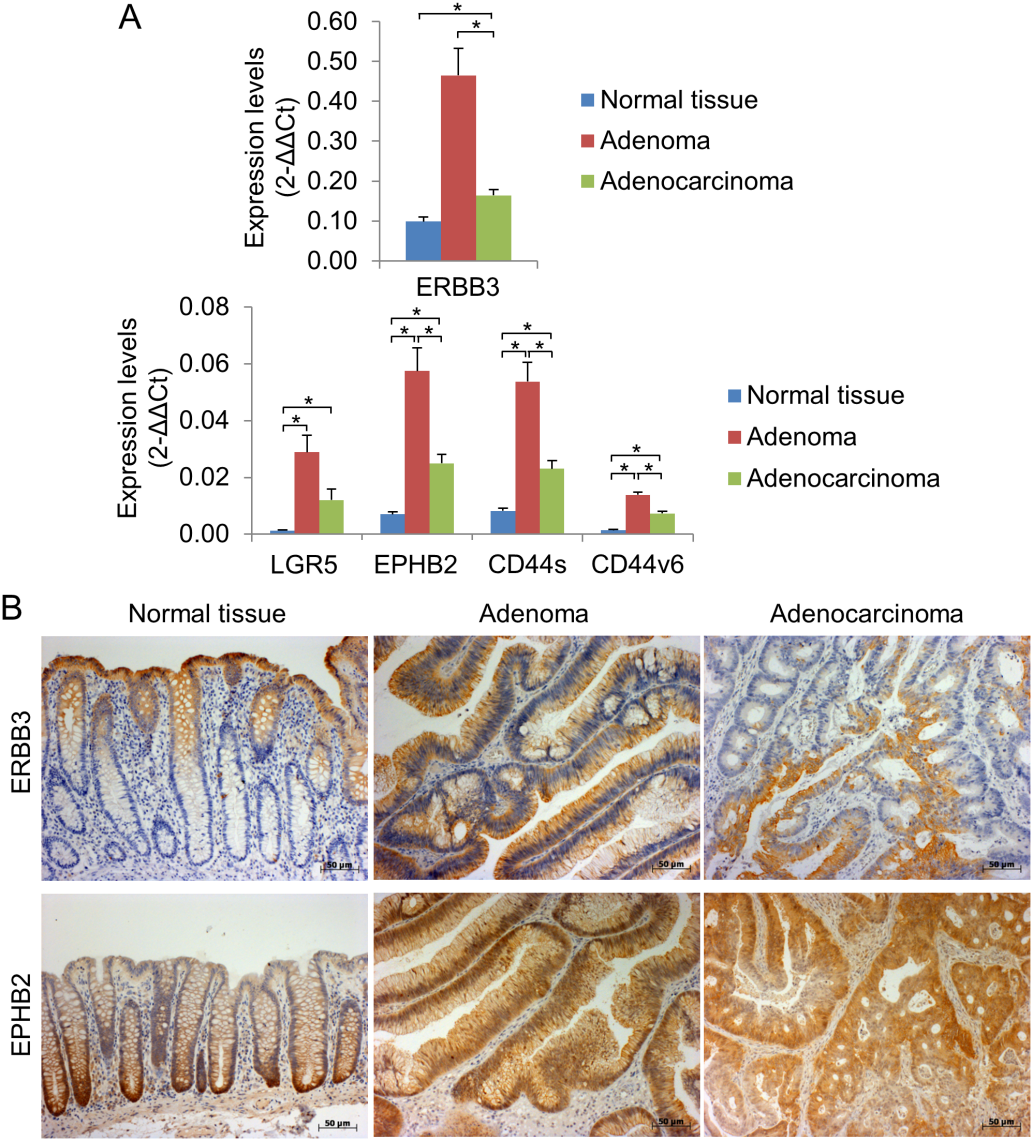
ERBB3 and intestinal stem cell markers LGR5, EPHB2, CD44s and CD44v6 are over-expressed in colorectal adenomas and adenocarcinomas. (A): The average expression levels (2^-ΔΔCt^) for each gene was calculated relative to beta-2-microglobulin and β-actin expression levels by qRT-PCR in normal colon tissues (n = 54), colorectal adenomas (n = 4) and colorectal adenocarcinomas (n = 54) (mean±s.e.m.). Significant differences (*) were observed compared to control tissues (Paired Student’s T test, p<0.001). (B): Immunohistochemical detection of ERBB3 and EPHB2 in normal colon tissue, adenoma and adenocarcinoma. Scale bar, 50μm.

The expression levels of ERBB3 in cancer tissues compared to matched normal tissues were highly variable, ranging from 0.06 to 60.2-fold change respectively (Fig 1). 38.8% (19/49) of tumours had increased ERBB3 expression levels (>2-fold) compared to matched normal tissues (Fig 1). The median level of ERBB3 expression in adenocarcinomas (0.141) was significantly higher than that in normal tissues (0.079) (Wilcoxon matched-pairs test, p<0.0005) (Fig 1). Similar results were obtained in adenomas compared to normal tissues, although the difference did not reach statistical significance (Paired Student’s T test, p = 0.061) probably due to the low number of adenoma samples in this patient cohort (n = 4) (Fig 2A). The mean value of the logarithm of the fold change (0.587) was significantly different from 0 (p = 0.009, one-sample t-test). Linear regression modelling indicated that ERBB3 expression levels were also significantly higher in stage IV tumours compared to stage I tumours (Coefficient = 1.36, (95% confidence interval (CI) 0.07, 2.64), p = 0.039, data not shown). There were no other significant relationships between ERBB3 expression levels and clinical markers such as disease stage, grade and TNM stages. Increased expression of ERBB3 in adenomas and adenocarcinomas compared to normal tissues was confirmed by immunohistochemistry (Fig 2B). ERBB3 was predominantly localised to the apical and basolateral cell surface of normal intestinal epithelial cells and was detected at the cell surface in the majority of tumours with some diffuse cytoplasmic staining apparent in a few cases (S1 Fig).

The expression of multiple intestinal stem cell markers was investigated (LGR5, EPHB2, CD44s and CD44v6). CD44s is an isoform common to all CD44 variants and has been used to identify putative stem cells in multiple tissue types. A recent study suggested that CD44v6, that includes variant exon 6, was an important regulator of intestinal stem cells and cancer formation so we have also analysed this in our studies [29, 30]. LGR5, EPHB2, CD44s and CD44v6 were all significantly up-regulated in cancer tissues compared to normal tissues (median increase 9.4-fold, 3.5-fold, 3.0-fold and 6.4-fold respectively, all p-values <0.0005; one-sample t-tests of logged values = 1) (Fig 1). Similar results were obtained in adenomas compared to normal tissues (Fig 2A). The expression levels of LGR5, EPHB2, CD44s and CD44v6 were increased at least 2-fold in 70.8% (34/48), 72.0% (36/50), 66.0% (33/50) and 86.0% (43/50) of tumour tissues compared to matched normal tissues (Fig 1). Increased expression of EPHB2 in adenomas and adenocarcinomas compared to normal tissues was confirmed by immunohistochemistry and was clearly localised to the epithelial tumour tissue (Fig 2B). EPHB2 expression levels were significantly higher in stage III, but not stage IV, tumours compared to stage I tumours (Coefficient = 1.47 (95% CI 0.30, 2.64), p = 0.015). There were no other significant relationships between the expression of stem cells and clinical markers.

### ERBB3 positively correlates with intestinal stem cell markers in colorectal cancer and colorectal cancer cell lines

Correlations between mRNA fold changes (adenocarcinoma *vs* normal) of markers described in Table 1 were assessed using Spearman rank correlation test. Intestinal stem cell marker fold changes (LGR5, EPHB2, CD44s and CD44v6) were positively inter-correlated (Table 1). Interestingly, positive correlations were found between ERBB3 and stem cell markers LGR5, EPHB2 and CD44v6 (Table 1). Similar results were obtained when adenoma and adenocarcinoma were combined (S3 Table).

**Table 1:** Correlations between markers in colorectal cancer (Spearman rank correlations).

		ERBB3	LGR5	EPHB2	CD44s
**LGR5**	rho	0.658			
	n	46			
	p-value	**<0.0001**			
**EPHB2**	rho	0.519	0.664		
	n	48	48		
	p-value	**0.0002**	**<0.0001**		
**CD44s**	rho	0.226	0.299	0.329	
	n	48	48	50	
	p-value	0.1228	**0.0395**	**0.0197**	
**CD44v6**	rho	0.478	0.465	0.486	0.480
	n	48	48	50	50
	p-value	**0.0006**	**0.0009**	**0.0003**	**0.0004**

**Fig 3:**
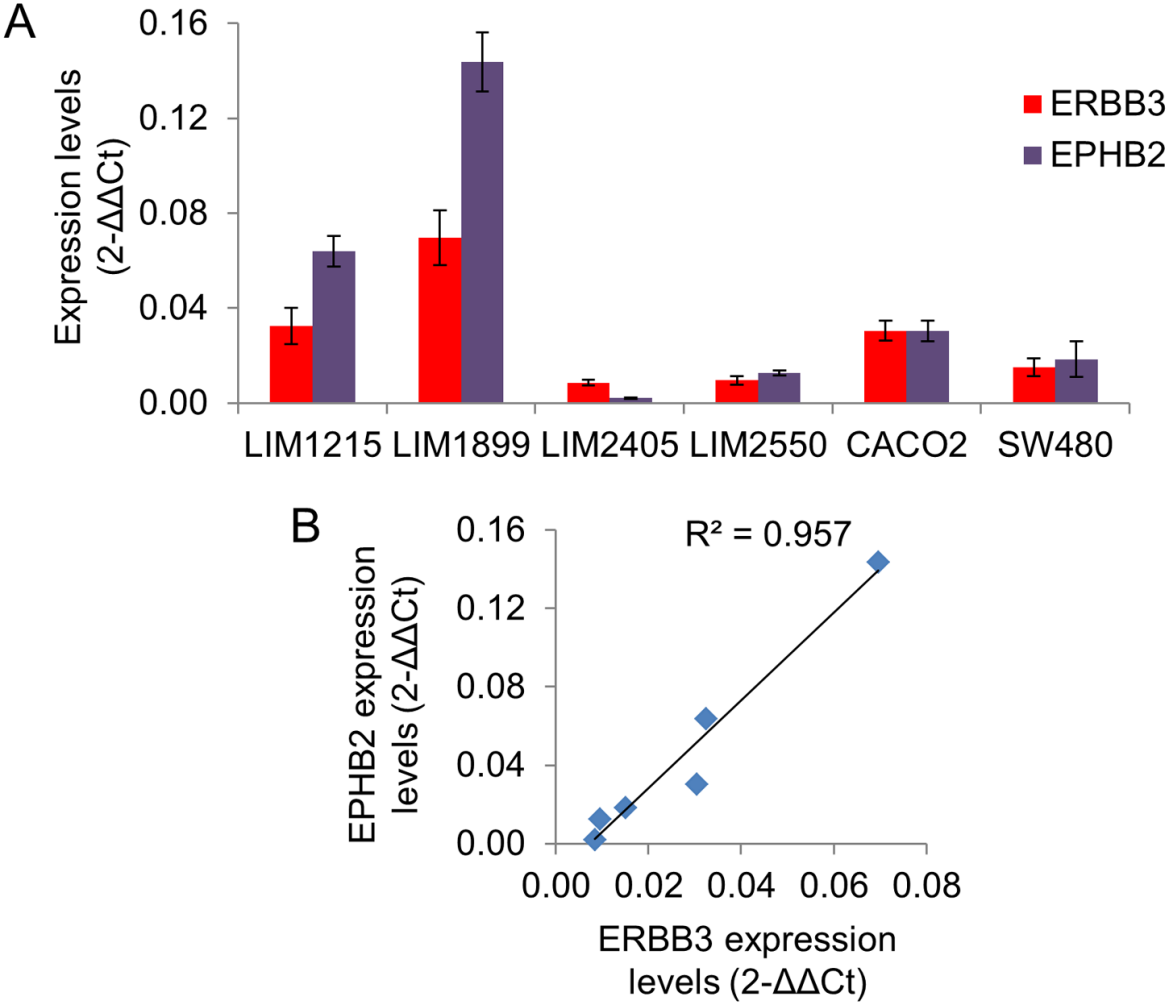
ERBB3 positively correlates with EPHB2 in colorectal cancer cell lines. (A): The average expression levels (2^-ΔΔCt^) of ERBB3 and EPHB2 were calculated relative to beta-2-microglobulin and β-actin expression levels by qRT-PCR in 6 different colorectal cancer cell lines (n = 3, mean±s.e.m.). (B): Positive linear correlation between ERBB3 and EPHB2 expression levels (Pearson correlation test, rho = 0.999, p<0.0001).

A qRT-PCR analysis of EPHB2 and ERBB3 expression levels was also conducted using 6 colorectal carcinoma cell lines. Both ERBB3 and EPHB2 were detected at various expression levels in these cell lines (Fig 3A). Most importantly, and as observed in the primary tumours, the expression of ERBB3 and EPHB2 was positively correlated (Fig 3B, Pearson correlation test, rho = 0.999, p<0.0001).

### Colorectal cancer resembles normal colon tissue

**Fig 4:**
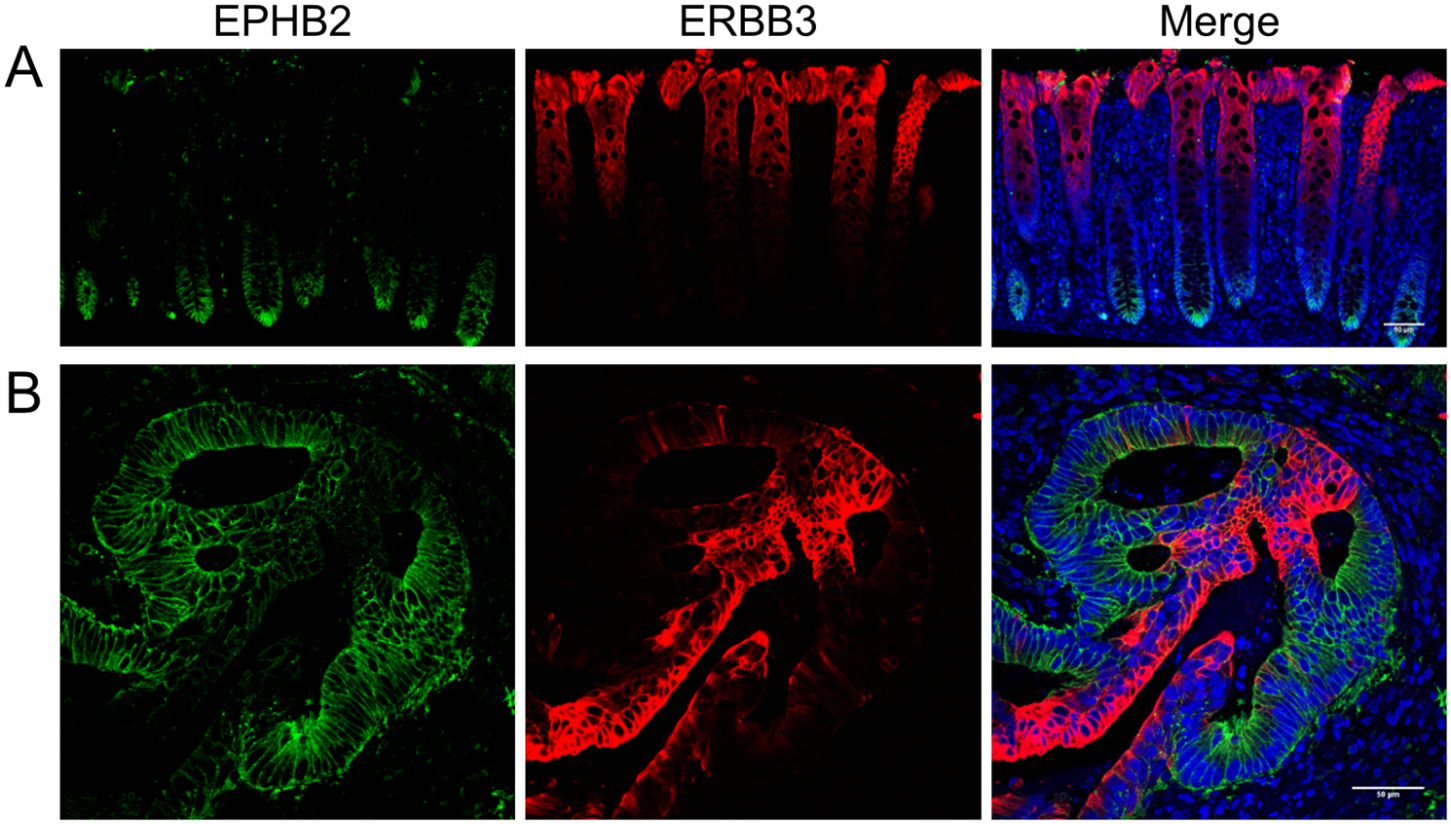
Colorectal tumours maintain tissue organisation similar to normal colon. Detection of EPHB2 (green) and ERBB3 (red, A and B) by co-immunofluorescence in normal colon (A) and colorectal cancer (B) (DAPI, blue). Scale bar, 50μm.

The positive correlations found at the RNA level between ERBB3 and intestinal stem cell markers prompted us to investigate the potential co-localisation of ERBB3 and intestinal stem cell markers in tumours. Multiple antibodies directed against LGR5 (Sigma-Aldrich, HPA012530; Abgent, AP2745; Abcam, ab75850), CD44 (Abcam, ab41478) and EPHB2 (R&D, AF467) were tested on normal human colon and colorectal cancer tissues. Only the polyclonal anti-EPHB2 antibody robustly detected EPHB2-positive cells at the bottom of normal human colonic crypts (n = 5, Fig 4A) where putative stem cells reside [3]. In contrast, ERBB3 positive cells were detected within the differentiated cell compartment at the top of crypts (Fig 4A). The expression patterns of ERBB3 and EPHB2 were mutually exclusive in normal colonic tissue. This pattern was also observed in adenocarcinomas where ERBB3 and EPHB2 marked distinct cell populations (n = 10) (Fig 4B).

These results suggest that although expression of ERBB3 and intestinal stem cell markers correlate with each other at the RNA level they do not mark the same cells within tumours.

### ERBB3 and EPHB2 mark distinct cell populations in colorectal cancer

**Fig 5:**
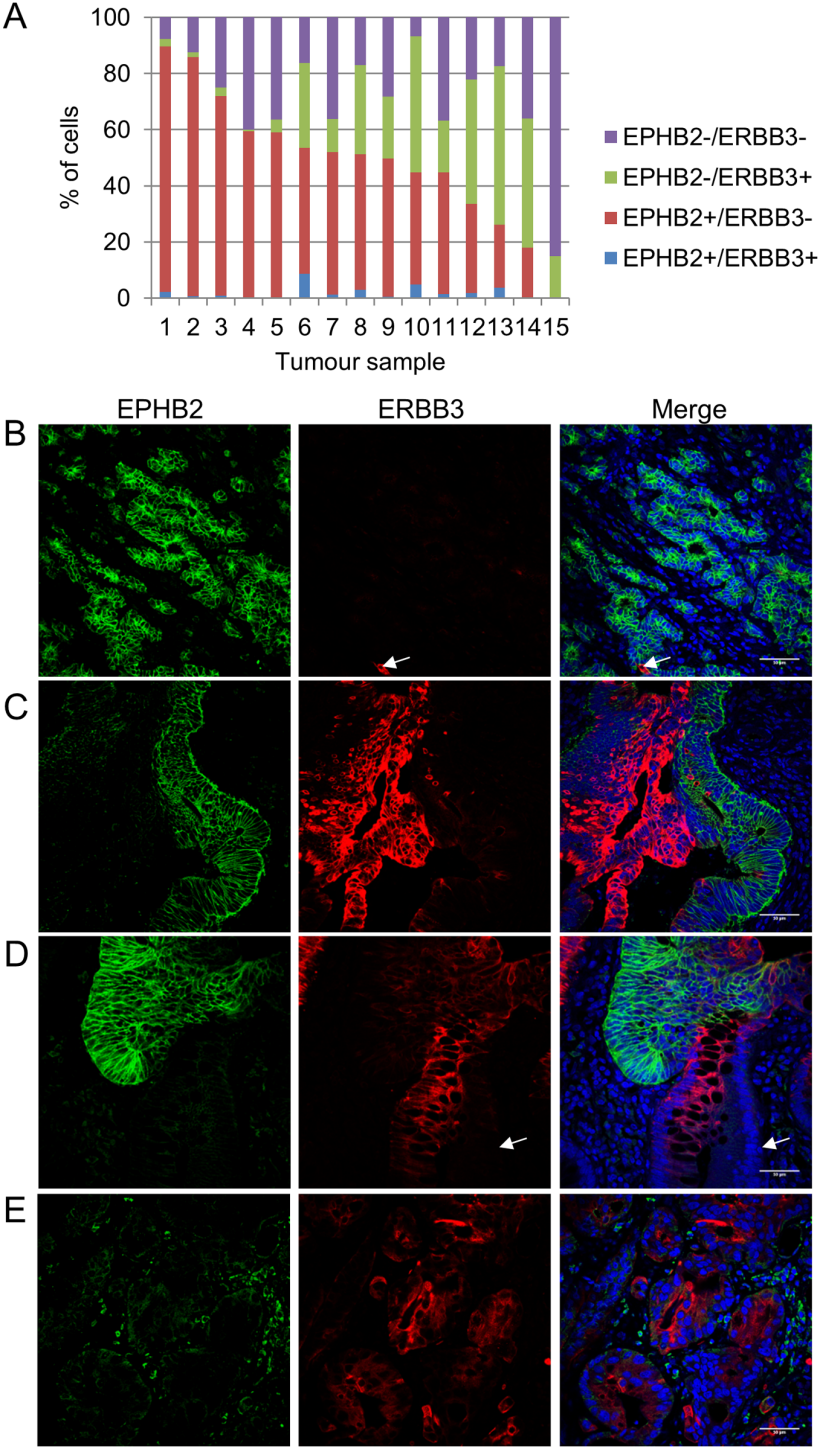
ERBB3 and EPHB2 mark distinct cell populations in colorectal cancer. (A): Frequency of EPHB2+ and ERBB3+ cells in colorectal cancer (n = 15). (B, C, D, E): Co-immunofluorescent detection of EPHB2 (green) and ERBB3 (red) in representative colorectal cancer samples counterstained with DAPI (blue). Note the presence of one EPHB2-/ERBB3+ cell (white arrow) in a tumour enriched for EPHB2+/ERBB3- cells (B). Note the presence of double negative cells (white arrow) in a tumour containing both EPHB2+/ERBB3- and EPHB2-/ERBB3+ cell populations (D). Scale bar, 50μm.

We sought to investigate whether the contrasting expression pattern of ERBB3 and EPHB2 was conserved in multiple tumours or if some tumours contained cells which co-expressed both markers. Following co-immunofluorescence and image analysis, the number of EPHB2+ and ERBB3+ cells was quantitated in 15 colorectal cancers (3500 cells per tumour). Three different types of tumours were identified based on their expression profiles. 33.3% (5/15) of tumours were enriched for EPHB2+ cells and lacked ERBB3+ cells (Fig 5A, tumours 1–5; Fig 5B). 60% (9/15) of tumours had reciprocal expression of EPHB2 and ERBB3 (Fig 5A, tumours 6–14; Fig 5C and 5D). One tumour was characterised by the absence of EPHB2+ cells and presence of a large population of double negative cells (EPHB2-/ERBB3-, 85.1% of cancer cells) (Fig 5A and 5E). Of note, all the tumours studied contained double negative cells (26.4% of tumour cells), ranging from 6.8% to 85.1% of cells per tumour. The presence of cells that co-expressed EPHB2 and ERBB3 was rare (2.1% tumour cells). The expression of ERBB3 was also investigated in 6 colorectal cancer cell lines by immunofluorescence (S2A Fig). In accordance with the qRT-PCR data, ERBB3 was detected in LIM1215, LIM1899, CACO2 and SW480 cancer cells. Importantly, the expression of ERBB3 was heterogeneous in these cell lines as marked by the presence of ERBB3+ and ERBB3- cells. In addition, in the cell lines that contained a detectable and robust ERBB3 cell population (LIM1215 and LIM1899), EPHB2 and ERBB3 marked distinct cell populations (S2B Fig), as observed in our study in human colorectal tumours. These results suggest that distinct cell populations are found in these cell lines.

In conclusion, our data suggest that EPHB2 and ERBB3 identify different sub-types of colorectal cancer, including stem cell enriched tumours (EPHB2+/ERBB3-), tumours that contain mutually exclusive compartments (EPHB2+/ERBB3+) or tumours lacking an EPHB2 stem cell compartment (EPHB2-).

### ERBB3+ cancer cells are predominantly non proliferative and differentiated in contrast to EPHB2+ cancer cells

**Fig 6:**
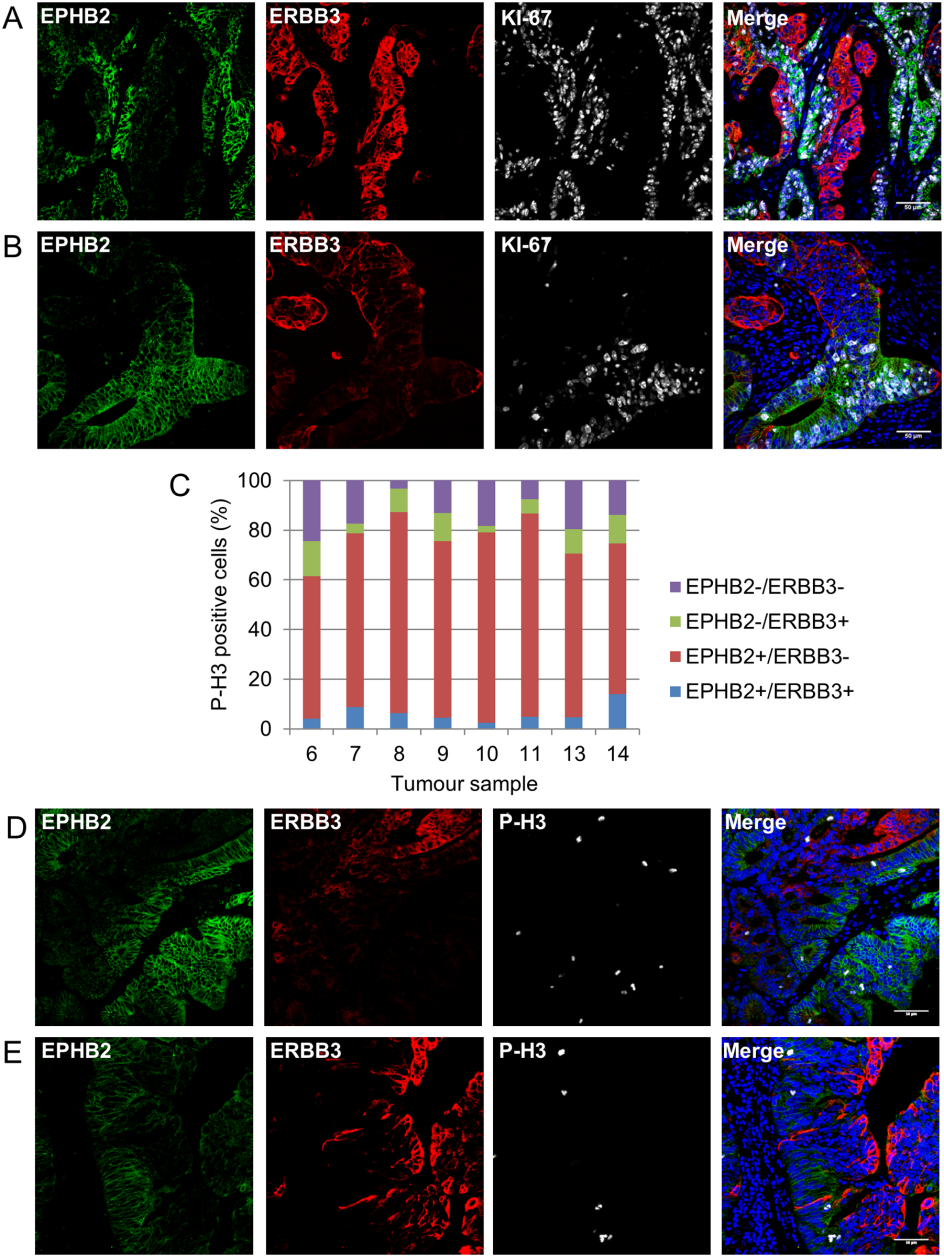
ERBB3+ colorectal cancer cells are predominantly non proliferative in contrast to EPHB2+ cells. (A and B): Detection of EPHB2 (green), ERBB3 (red) and KI-67 (grey) by co-immunofluorescence in two representative colorectal cancer samples (DAPI, blue). (C): Distribution of P-H3+ proliferative cells within the 4 distinct cell populations in 8 colorectal cancer samples. (D and E) Detection of EPHB2 (green), ERBB3 (red) and P-H3 (grey) by co-immunofluorescence in two representative colorectal cancer samples (DAPI, blue). Scale bar, 50μm.

To define the proliferative status of ERBB3+ cancer cells in primary colorectal cancer, tissues were triple stained for KI-67, a known cell proliferation marker, ERBB3 and EPHB2. Surprisingly, ERBB3+ cancer cells rarely co-localised with KI-67 nuclear staining (Fig 6A and S3 Fig). In contrast, in the same tumour, EPHB2+ cells were mainly positive for KI-67 (Fig 6A). Double negative cells (EPHB2-/ERBB3-) were also KI-67 positive. Similar observations were found in multiple tumours (Fig 6B and S4 Fig). Interestingly, these observations were reminiscent of the proliferative status of normal colon since the KI-67+ proliferative cells were only found at the lower part of the crypt where EPHB2+ cells are localised (S5 Fig).

To confirm the non-proliferative nature of EPHB2-/ERBB3+ cells, a second proliferation marker (P-H3), which detects mitotic cells, was used on 8 colorectal cancer samples that contained a strong ERBB3+ cell population (based on Fig 4A). Following co-immunofluorescence and image analysis, the number of P-H3+ cells in the four different cell populations (EPHB2+/ERBB3+; EPHB2+/ERBB3-; EPHB2-/ERBB3+; EPHB2-/ERBB3-) was quantitated (between 100–200 P-H3+ cells per tumour). 70.0% (ranging from 57.5% to 81.8%) of P-H3+ cells were located in the EPHB2+/ERBB3- population, while in contrast only 8.8% (2.5–14.0%) of P-H3+ cells were located in the EPHB2-/ERBB3+ cell population (Fig 6C). P-H3+ cells were rarely double positive for EPHB2 and ERBB3 (5.6%) (Fig 6C). Representative images showing localisation of P-H3+ cells in the EPHB2+/ERBB3- cell population in 2 different tumours are presented in Fig 6D and 6E.

**Fig 7:**
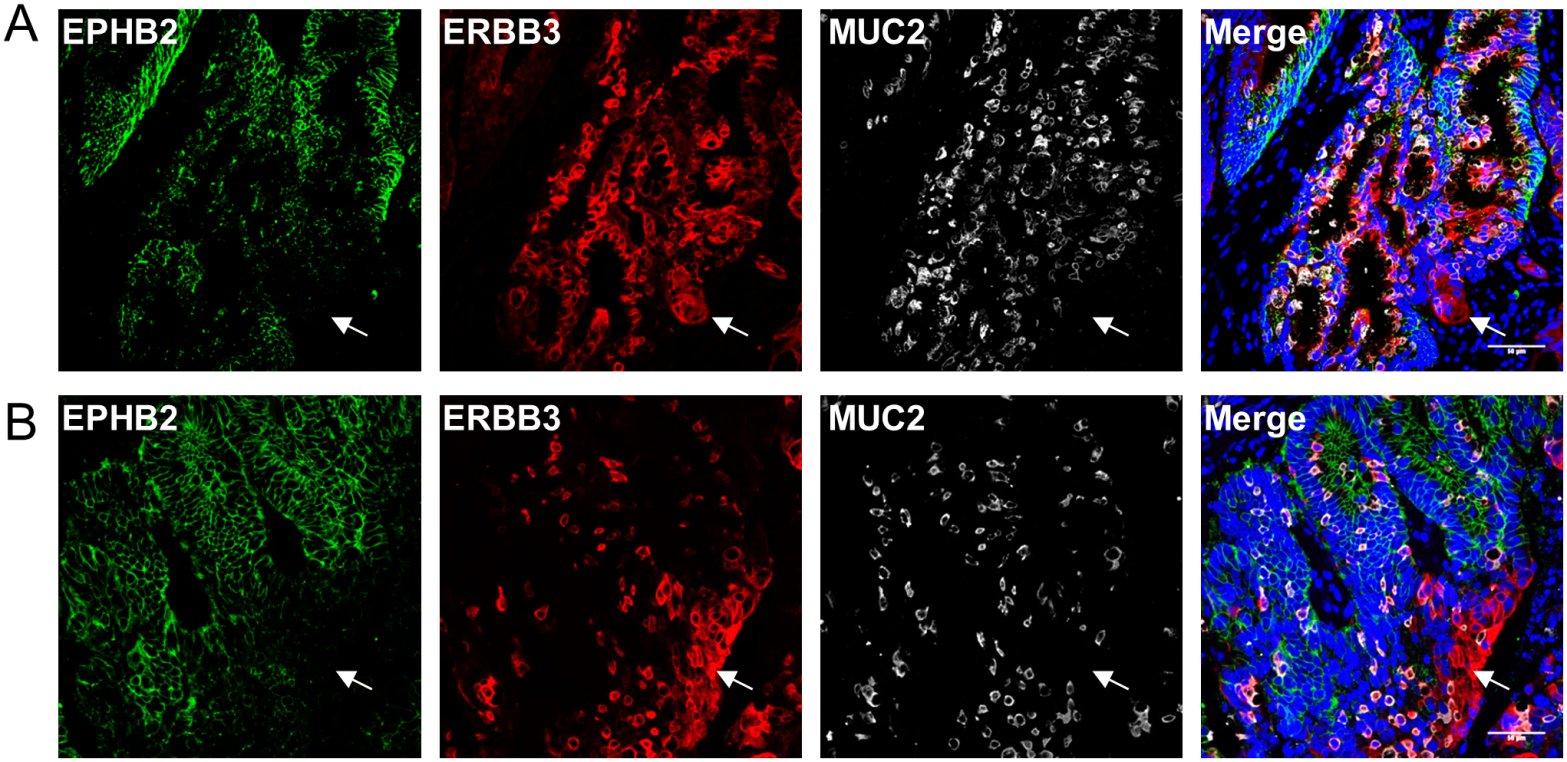
MUC2+ differentiated colorectal cancer cells are predominantly ERBB3+. Detection of EPHB2 (green), ERBB3 (red) and MUC2 (grey) by co-immunofluorescence in two representative colorectal cancer samples (DAPI, blue). Note the presence of EPHB2-/ERBB3+ cells (white arrow) that are MUC2-. Scale bar, 50μm.

To investigate the differentiated status of EPHB2-/ERBB3+ cells in colorectal cancer, tissues were triple stained for MUC2, a marker of differentiated goblet cells, ERBB3 and EPHB2. Interestingly, most MUC2+ colorectal cancer cells were also EPHB2-/ERBB3+ (Fig 7A). Similar observations were found in multiple tumours (Fig 7B and S6 Fig). However, a sub-group of EPHB2-/ERBB3+ cancer cells were not positive for MUC2 in these tumours (Fig 7A and 7B, white arrows). In addition, colorectal tumours that did not contain any MUC2+ cells still had an EPHB2-/ERBB3+ cell population (data not shown).

Taken together, these data demonstrate that ERBB3+ colorectal cancer cells are predominantly non proliferative and differentiated, in contrast to EPHB2+ cells.

## Discussion

In order to define whether ERBB3 signalling plays a role in the regulation of colorectal cancer stem cells, the expression of ERBB3 and intestinal stem cell markers were investigated in multiple colorectal tumours, including early stage adenomas and carcinomas (Stage I-IV). We found significant elevation of ERBB3 mRNA levels in adenomas and colorectal cancers compared to normal tissues. Similar results were obtained for LGR5, EPHB2, CD44s and CD44v6, in accordance with previous studies.[4, 5, 8] Taken together, these data suggest that the expression of ERBB3 and stem cell markers are elevated throughout the progressive stages of carcinogenesis. Most interestingly, we determined that ERBB3 and several molecules which mark stem cell populations positively correlate at the RNA level in colorectal cancer.

This finding prompted us to investigate if ERBB3 was present in the stem cell population within tumours. We analysed whether EPHB2, a defined stem cell marker, co-localised with ERBB3 expression in colorectal cancer tissues. This revealed three different cancer subsets based on i) enrichment of EPHB2+ cells and lack of ERBB3+ cells, ii) absence of EPHB2+ cells or iii) the presence of both ERBB3+ and EPHB2+ cancer cell populations. In the later subset, ERBB3 and EPHB2 marked different mutually exclusive cell populations with distinct proliferative potentials, the majority of ERBB3+ cells being non-proliferative. Surprisingly, these results revealed that although there was a positive correlation between expression of ERBB3 and EPHB2 at the RNA level, these two markers were not present in the same cells. Interestingly, expression of ERBB3 and EPHB2 in distinct domains was detected in normal colonic epithelium where EPHB2 labelled stem cells and proliferative progenitors at the crypt bottom and ERBB3 marked the non-proliferative differentiated cell compartment at the top of intestinal crypts. Our results show that most colorectal cancers we examined resemble normal tissue with distinct cell compartments and proliferative status and that a dual EPHB2/ERBB3 signature could identify tumours with this morphology. A similar pattern of expression has been observed in colorectal cancers using EPHB2 and cytokeratin 20 as a differentiation marker [8, 31]. Interestingly, cytokeratin 20 expression levels were down-regulated in colorectal cancer tissues compared to normal tissues, in contrast to ERBB3 in our studies, which suggests a more complex role for ERBB3 [8]. Consistent with the expression profile of ERBB3, the expression levels of PMEPA1, which is a marker of terminally differentiated cells exclusively found at the surface of colonic epithelial crypts, are increased in colorectal tumours compared to normal tissues [32], suggesting that molecules that mark differentiated cell population can also be elevated in some cases during colorectal carcinogenesis.

Our results indicate that targeting ERBB3 in colorectal cancer using monoclonal antibodies, that are currently under development [13], may target differentiated cells and contribute to the elimination of the tumour bulk but may not affect the proliferative cancer stem-like cell population. This remains to be experimentally tested. The destruction of differentiated colorectal cancer cells may be beneficial as these cells have been recently described as important mediator of resistance to therapy [33] and may protect tumour initiating cells from the chemotherapy agent irinotecan due to their drug-expelling capacities [33]. It would be interesting to define whether the differentiated cell population described by Emmink *et al*. overlaps with the ERBB3+ differentiated population. In addition, these non-proliferative ERBB3+ cells may still have a role to play during tumour recurrence as it has been reported that post-mitotic intestinal cells can de-differentiate, acquire stem cell like properties and initiate tumourigenesis in some cases [34].

Elevated levels of ERBB3 expression have been associated with decreased patient survival in colorectal cancer [20, 21]. It seems crucial to define whether these ERBB3+ cells are important drivers of carcinogenesis and whether they act by supporting the associated stem cell population or directly contributing to a decrease in patient survival.

In summary, our results show that elevated expression of ERBB3 and intestinal stem cell markers are common features of colorectal cancers and identify tumours that contain differentiated non-proliferative cell populations distinct from proliferative regions where cancer stem cells reside. Based on the expression of EPHB2 stem cell marker and the ERBB3 differentiation marker, we tentatively propose that colorectal cancers are organised into i) stem-like cell enriched tumours, ii) differentiated tumours with a stem-like cell compartment or iii) lacking an EPHB2 stem cell compartment.

## Supporting Information

S1 Fig ERBB3 is predominantly located at the membrane in adenocarcinomas. Immunohistochemical detection of ERBB3 in 7 colorectal cancer samples showing a predominant strong membrane staining (A-D) or both diffuse cytoplasmic and membrane staining (E-G). Scale bar, 50μm.
(TIF)

S2 Fig ERBB3 is heterogeneously expressed in colorectal cancer cell lines. (A): Immunofluorescent detection of ERBB3 (red) in 6 different cancer cell lines counterstained with DAPI (blue). (B): Expression of ERBB3 (green) and EPHB2 (red) in LIM1215 and LIM1899 cancer cell lines counterstained with DAPI (blue). Scale bar, 50μm.
(TIF)

S3 Fig ERBB3 positive colorectal cancer cells are predominantly non-proliferative. Co-immunofluorescent detection of KI-67 (green) and ERBB3 (red) in two different colorectal cancer samples counterstained with DAPI (blue). Scale bar, 50μm.
(TIF)

S4 Fig ERBB3 positive colorectal cancer cells are predominantly non proliferative in contrast to EPHB2 positive cells. Co-immunofluorescent detection of KI-67 (green), ERBB3 (red, A, C, E) and EPHB2 (red, B, D, F) in three different colorectal cancer samples, DAPI (blue). Scale bar, 50μm.
(TIF)

S5 Fig EPHB2 positive cells are KI-67 positive in normal human colon. Co-immunofluorescent detection of EPHB2 (green) and KI-67 (red) in normal colon tissue (DAPI, blue). Note the absence of KI-67 positive cells in the differentiated compartment (white arrow). Scale bar, 50μm.
(TIF)

S6 Fig MUC2 positive cells are predominantly ERBB3 positive in human colorectal cancer. Co-immunofluorescent detection of EPHB2 (green), ERBB3 (red) and MUC2 (grey) in three different colorectal cancer samples, DAPI (blue). Scale bar, 50μm.
(TIF)

S1 Table List of primers used in this study. (TIF)

S2 Table Patients characteristics. (TIF)

S3 Table Correlations between markers in adenomas and colorectal cancers (Spearman rank correlations). (TIF)
