# Supplementary figures and images for: ERBB3 Positively Correlates with Intestinal Stem Cell Markers but Marks a Distinct Non Proliferative Cell Population in Colorectal Cancer
Source: PLoS One. 2015 Sep 14;10(9):e0138336. doi: 10.1371/journal.pone.0138336 (PMC4569358; doi:10.1371/journal.pone.0138336)

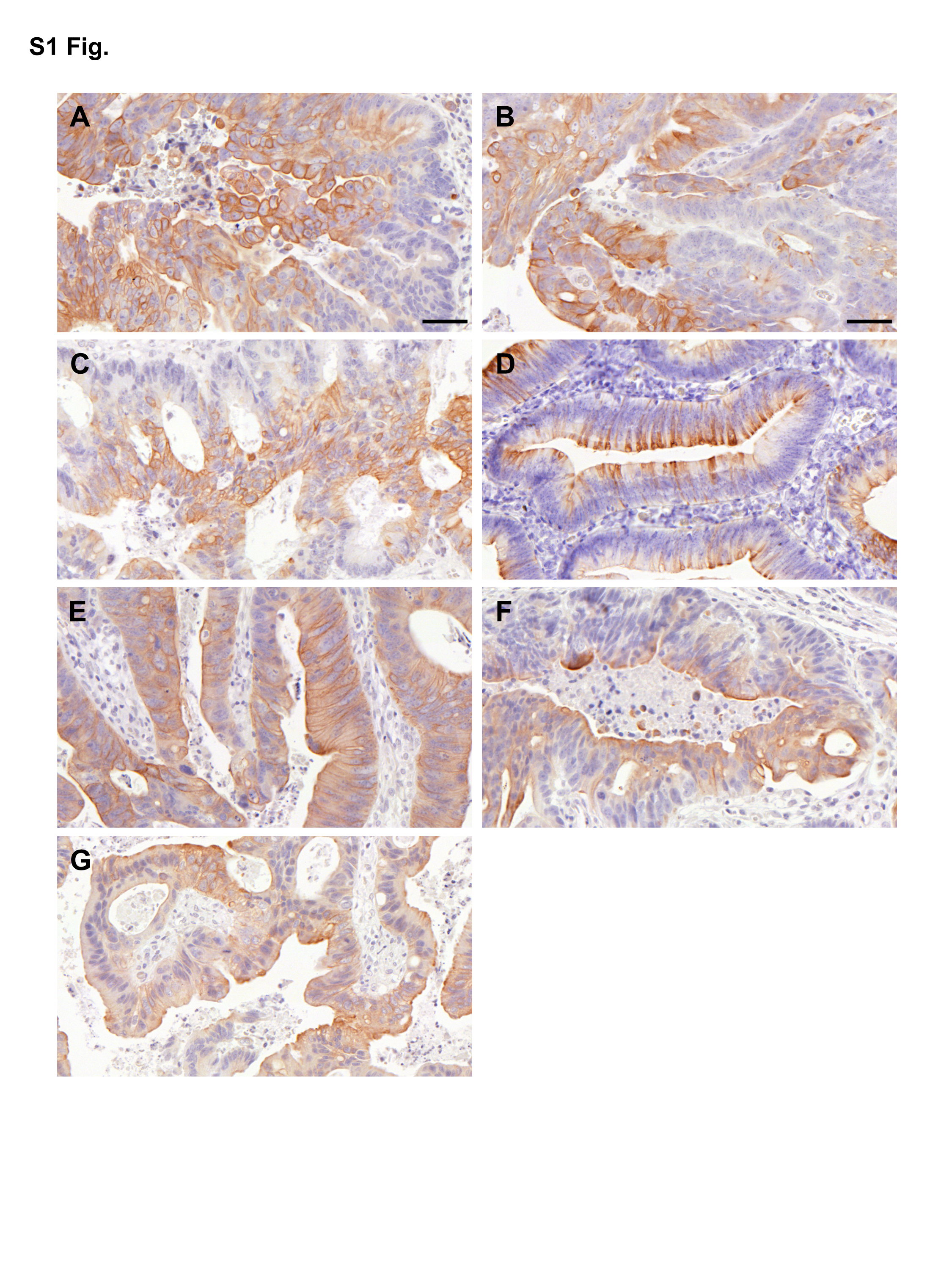

Supplement: S1 Fig — Immunohistochemical detection of ERBB3 in 7 colorectal cancer samples showing a predominant strong membrane staining (A-D) or both diffuse cytoplasmic and membrane staining (E-G). Scale bar, 50μm. (TIF) [file pone.0138336.s001.TIF]

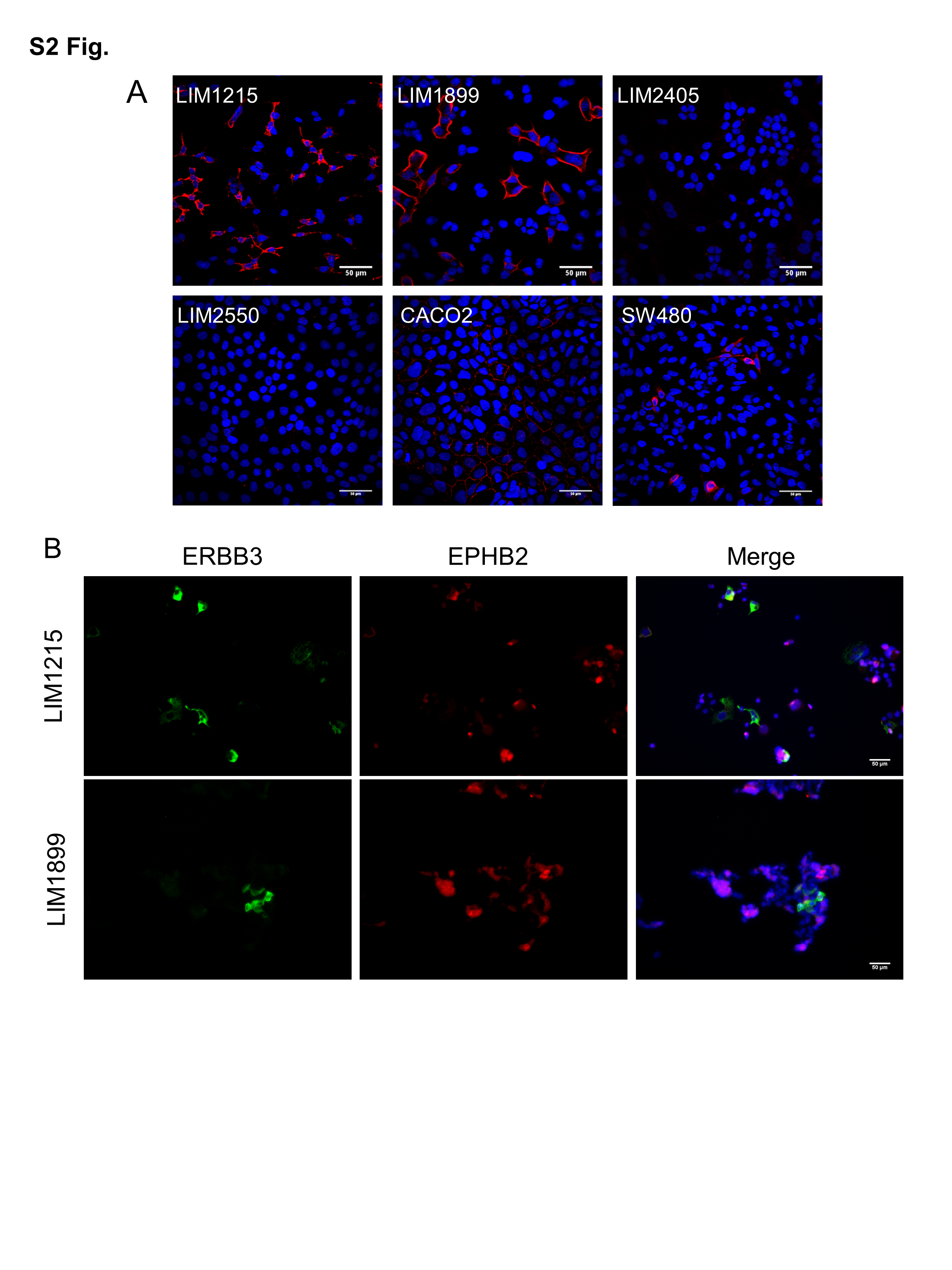

Supplement: S2 Fig — (A): Immunofluorescent detection of ERBB3 (red) in 6 different cancer cell lines counterstained with DAPI (blue). (B): Expression of ERBB3 (green) and EPHB2 (red) in LIM1215 and LIM1899 cancer cell lines counterstained with DAPI (blue). Scale bar, 50μm. (TIF) [file pone.0138336.s002.tif]

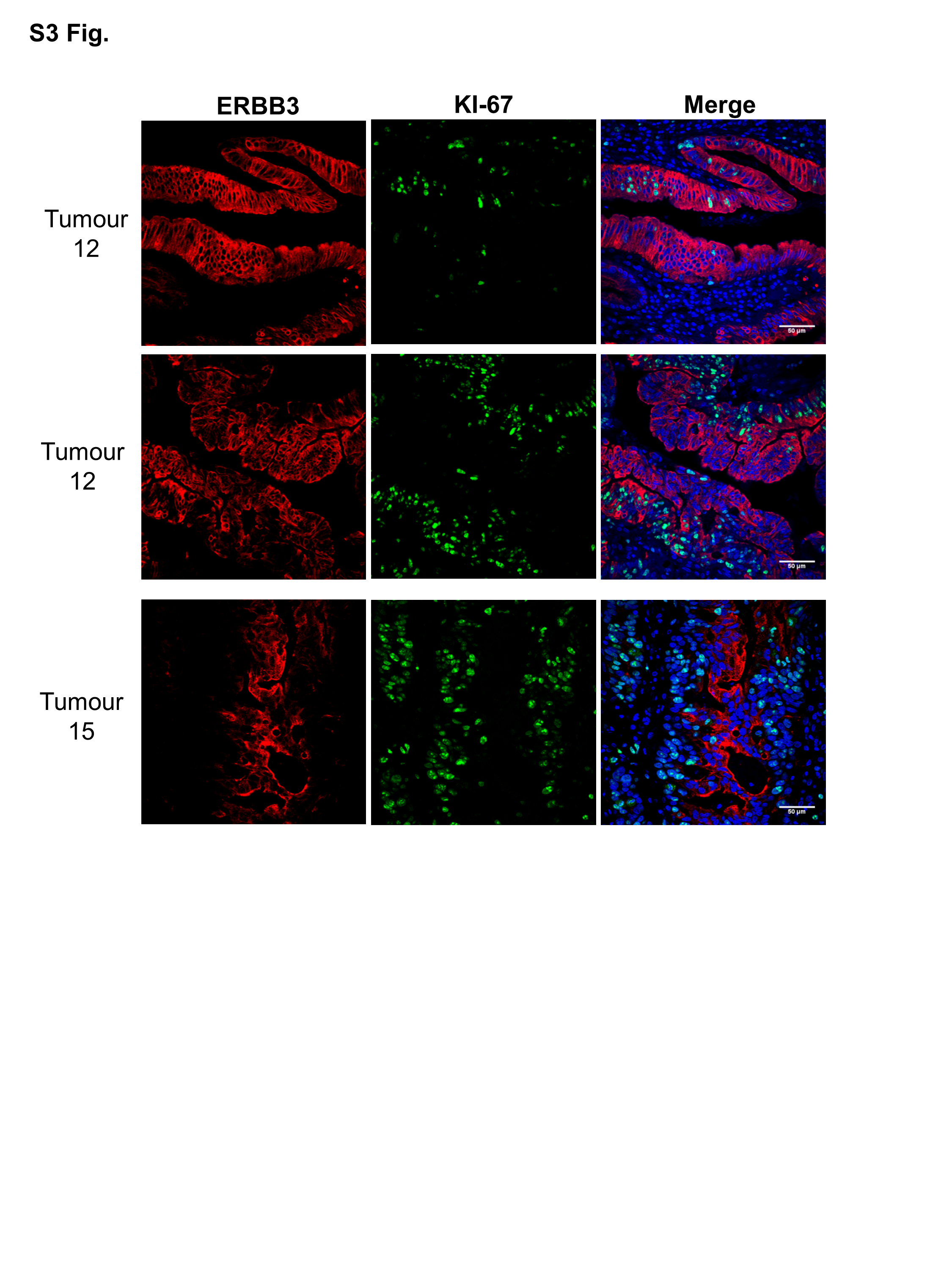

Supplement: S3 Fig — Co-immunofluorescent detection of KI-67 (green) and ERBB3 (red) in two different colorectal cancer samples counterstained with DAPI (blue). Scale bar, 50μm. (TIF) [file pone.0138336.s003.TIF]

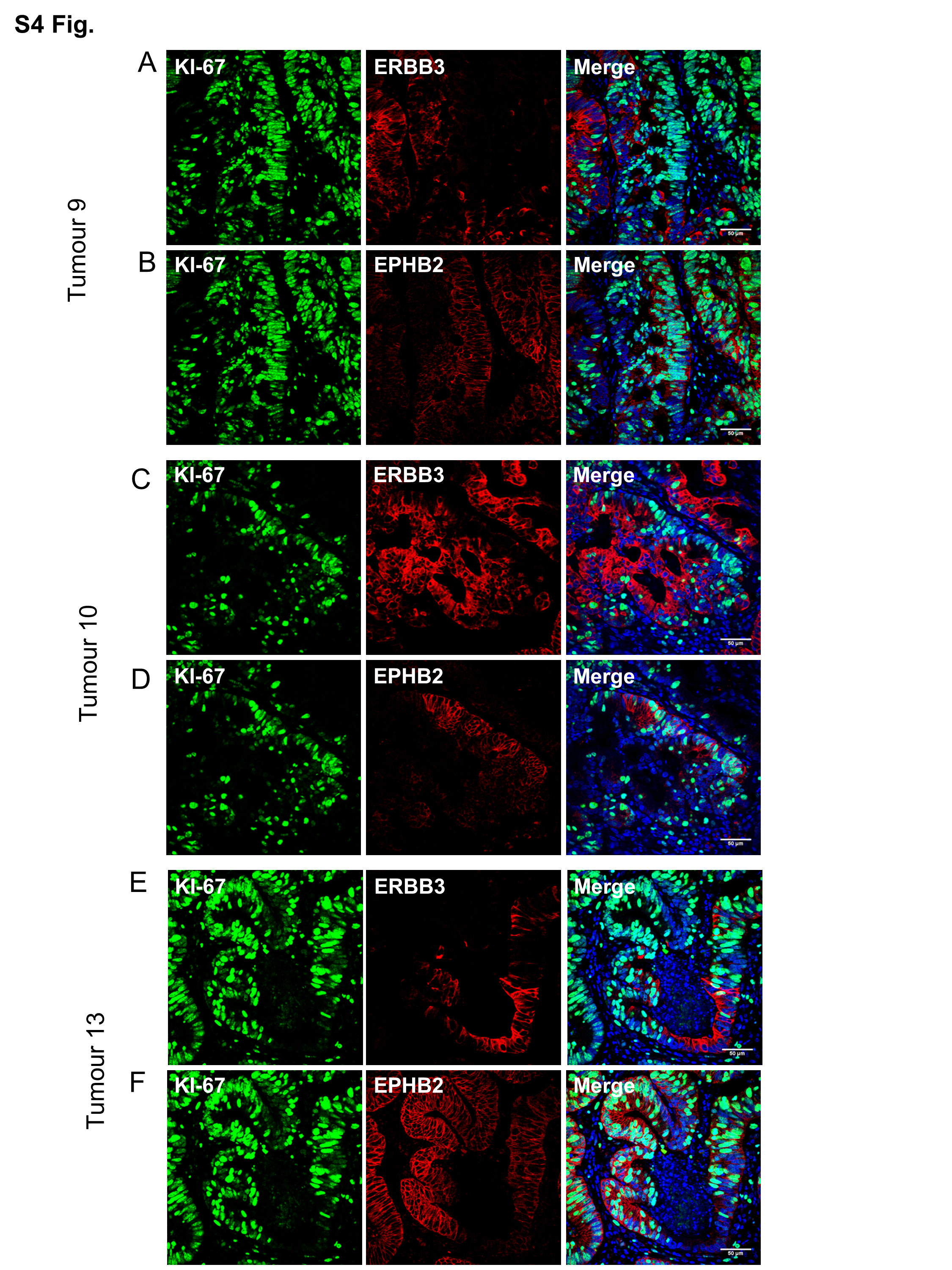

Supplement: S4 Fig — Co-immunofluorescent detection of KI-67 (green), ERBB3 (red, A, C, E) and EPHB2 (red, B, D, F) in three different colorectal cancer samples, DAPI (blue). Scale bar, 50μm. (TIF) [file pone.0138336.s004.TIF]

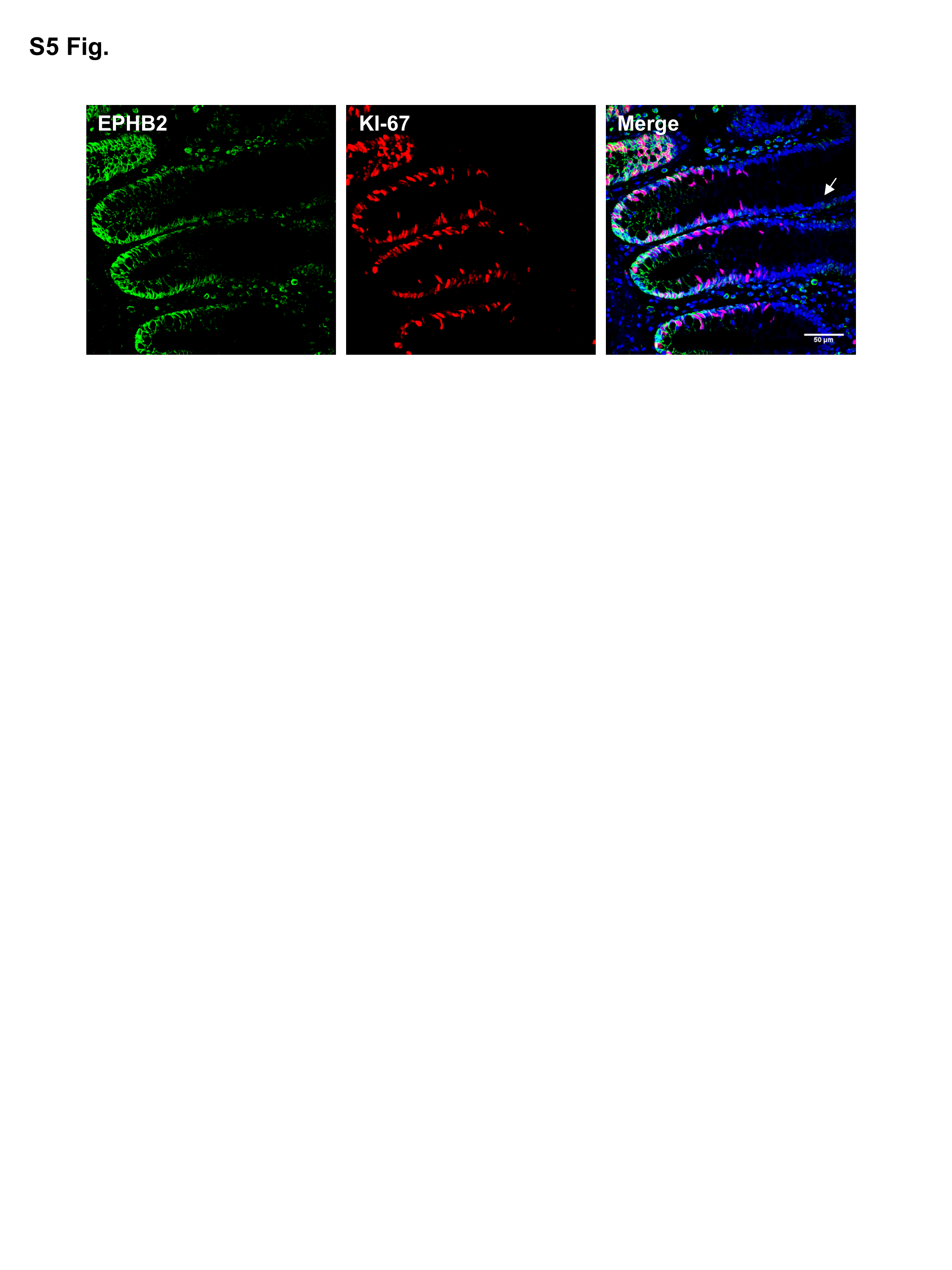

Supplement: S5 Fig — Co-immunofluorescent detection of EPHB2 (green) and KI-67 (red) in normal colon tissue (DAPI, blue). Note the absence of KI-67 positive cells in the differentiated compartment (white arrow). Scale bar, 50μm. (TIF) [file pone.0138336.s005.TIF]

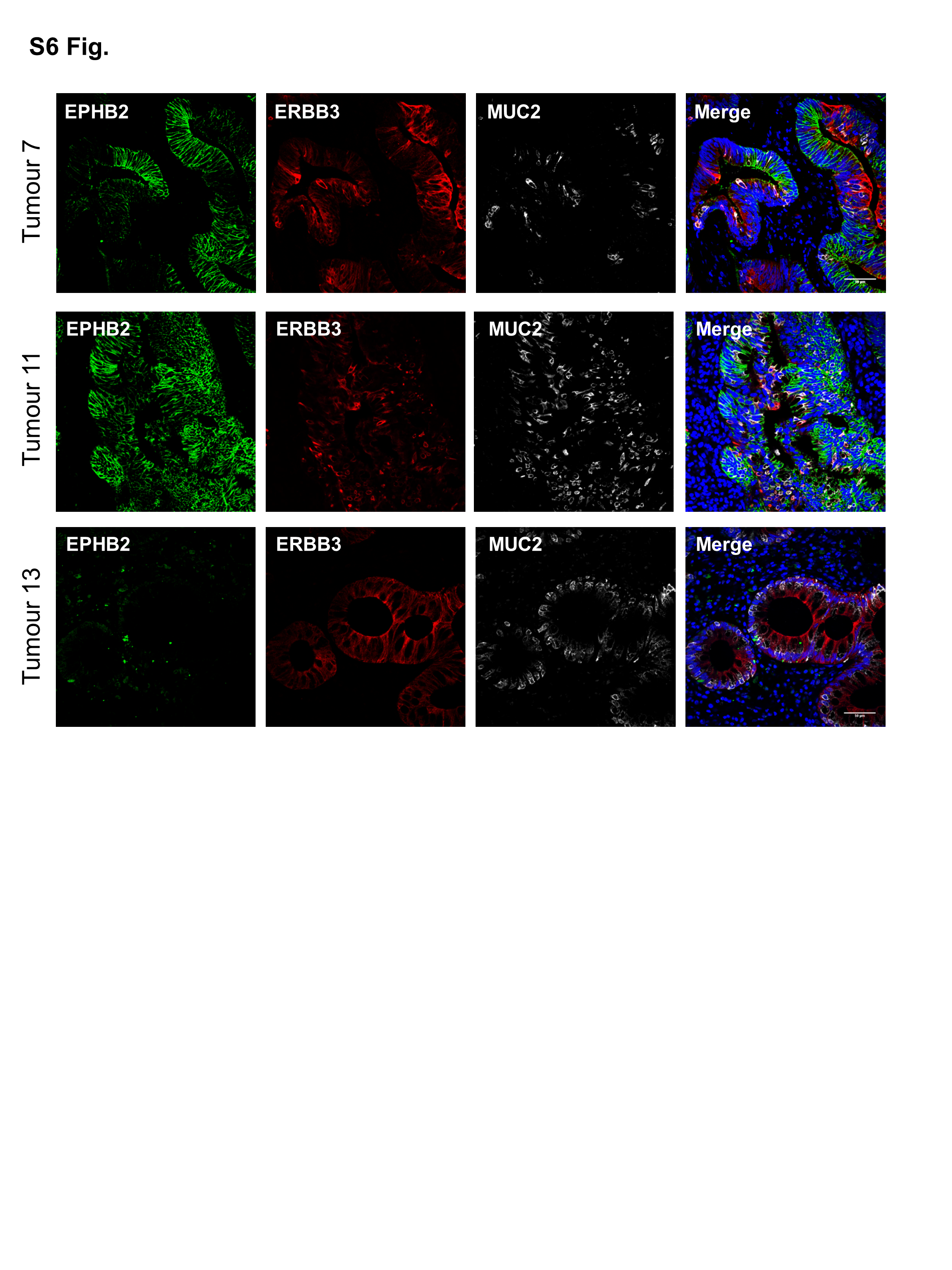

Supplement: S6 Fig — Co-immunofluorescent detection of EPHB2 (green), ERBB3 (red) and MUC2 (grey) in three different colorectal cancer samples, DAPI (blue). Scale bar, 50μm. (TIF) [file pone.0138336.s006.TIF]

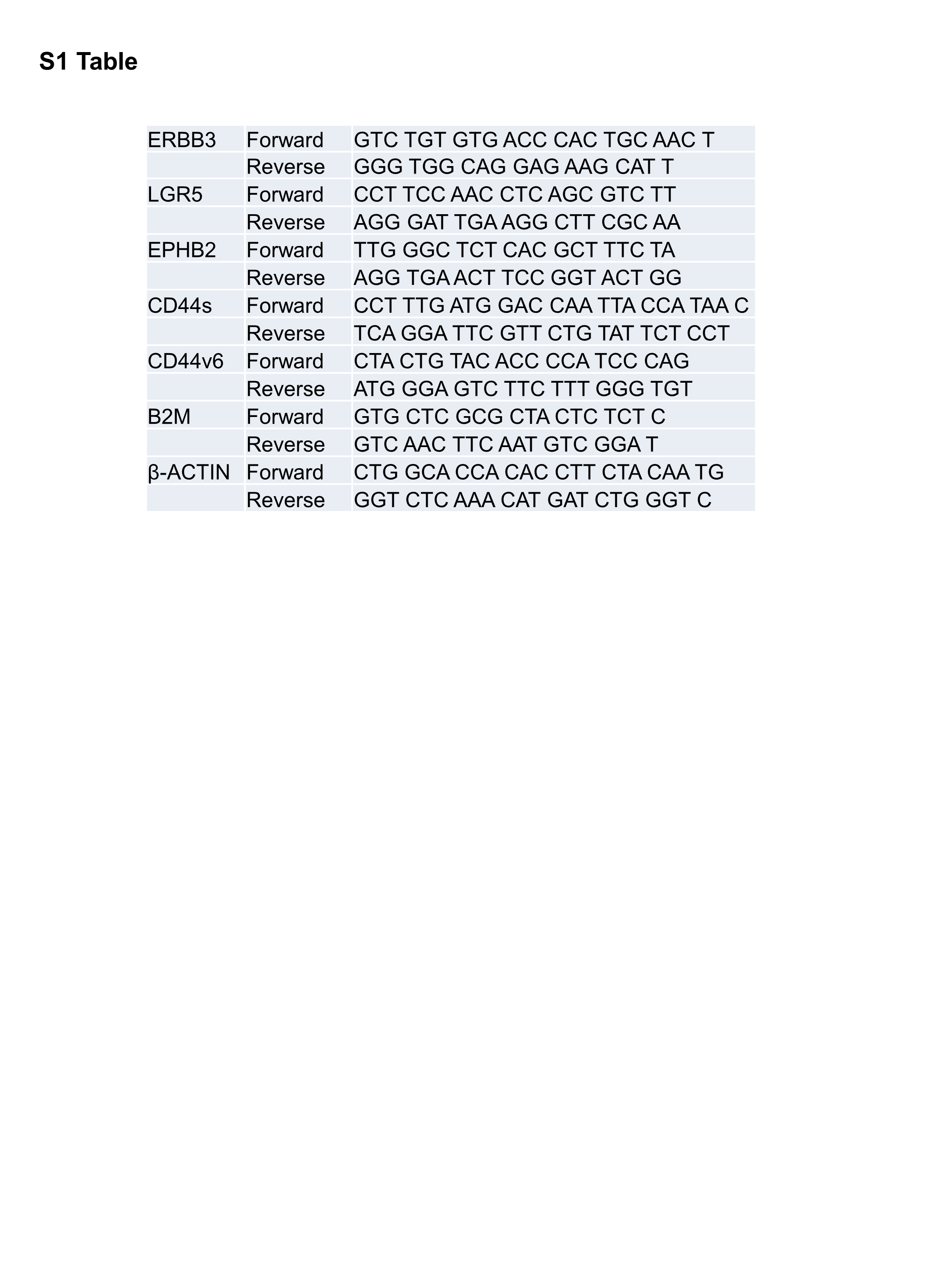

Supplement: S1 Table — (TIF) [file pone.0138336.s007.TIF]

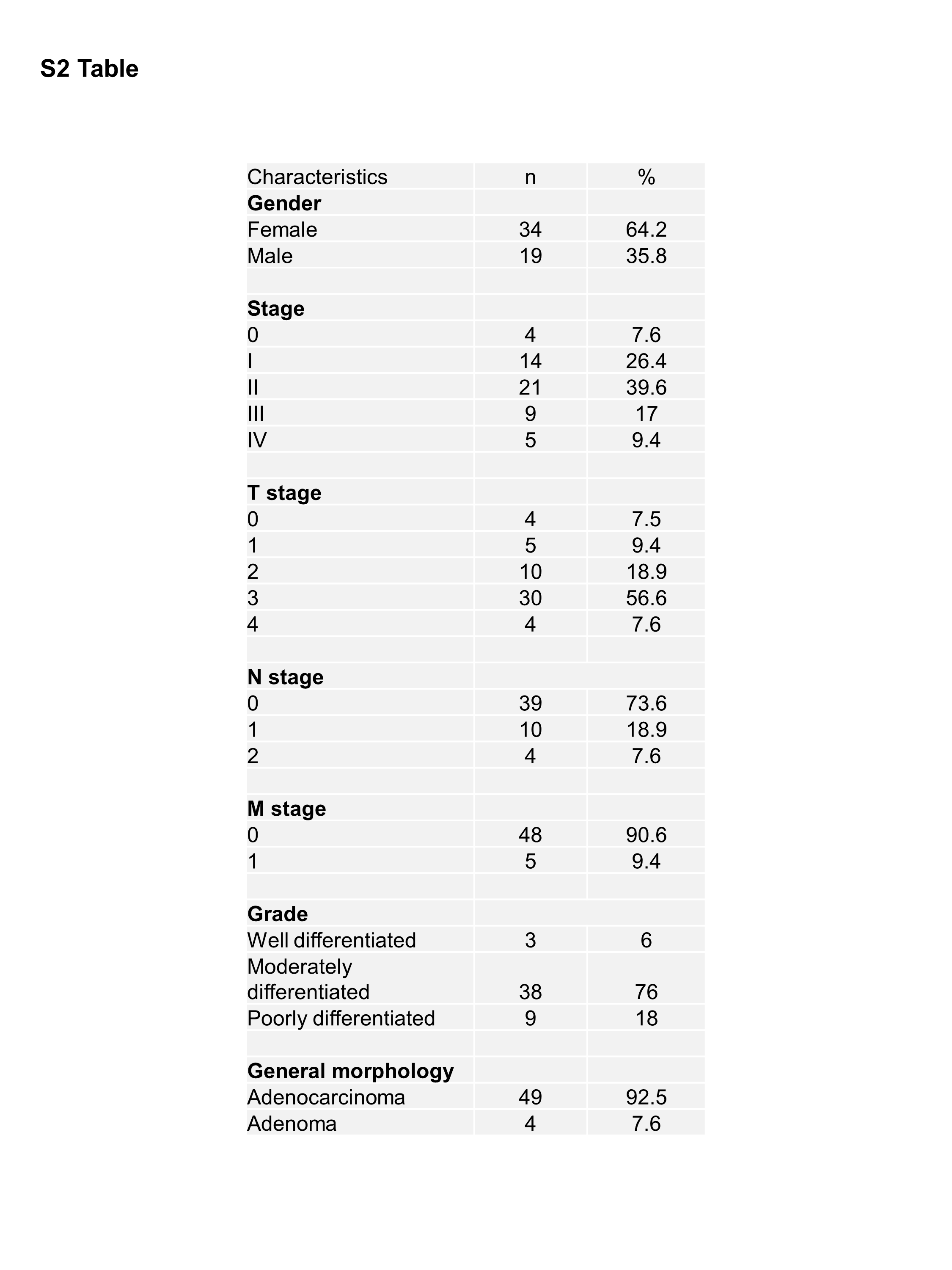

Supplement: S2 Table — (TIF) [file pone.0138336.s008.TIF]

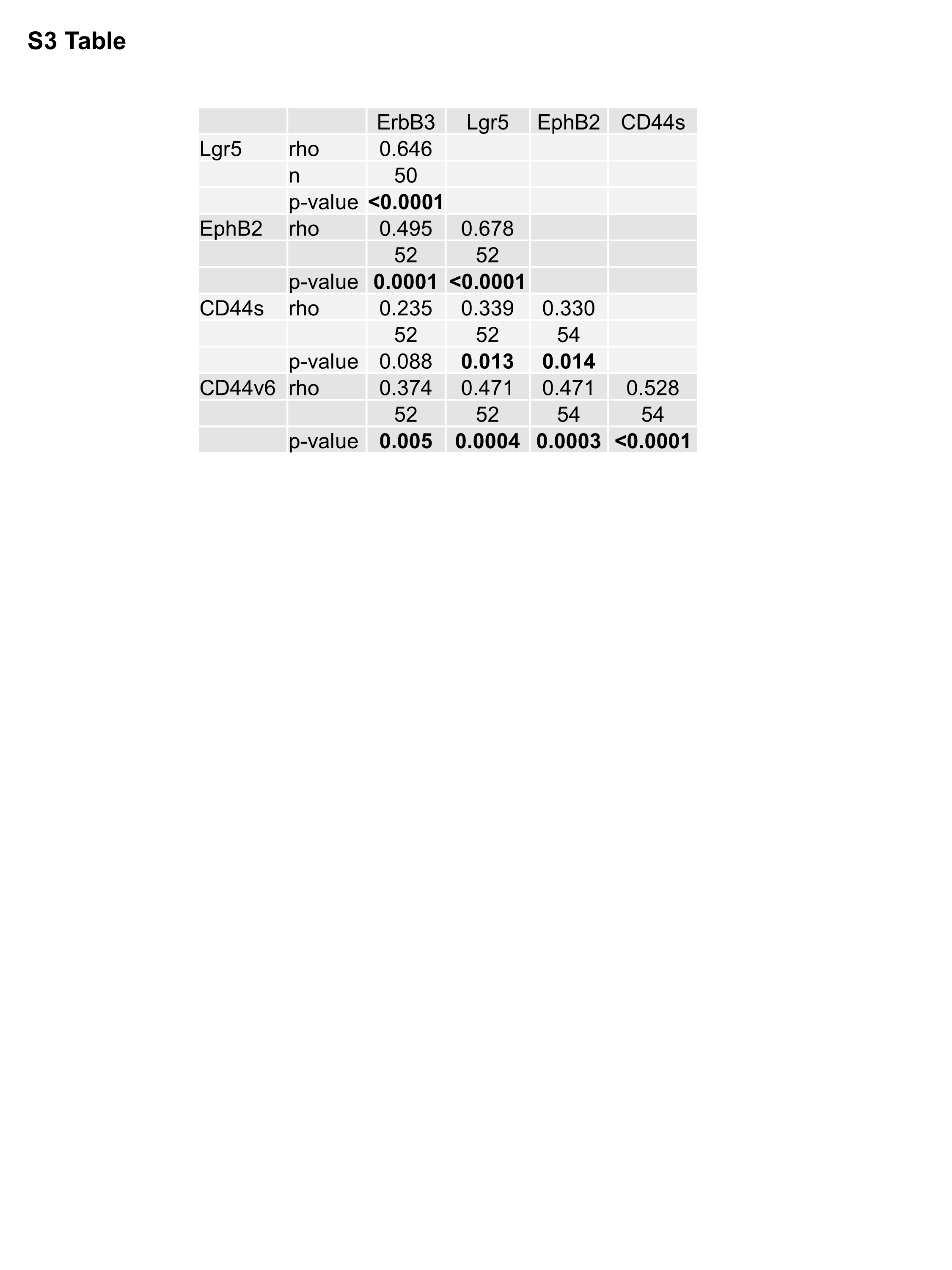

Supplement: S3 Table — (TIF) [file pone.0138336.s009.TIF]
